# Unravelling the Paradox of Loss of Genetic Variation during Invasion: Superclones May Explain the Success of a Clonal Invader

**DOI:** 10.1371/journal.pone.0097744

**Published:** 2014-06-10

**Authors:** Valerie Caron, Fiona J. Ede, Paul Sunnucks

**Affiliations:** 1 School of Biological Sciences, Monash University, Clayton, Victoria, Australia; 2 Biosciences Research Division, Department of Environment and Primary Industries, Bundoora, Victoria, Australia; Institute of Vegetables and Flowers, Chinese Academy of Agricultural Science, China

## Abstract

Clonality is a common characteristic of successful invasive species, but general principles underpinning the success of clonal invaders are not established. A number of mechanisms could contribute to invasion success including clones with broad tolerances and preferences, specialist clones and adaptation *in situ*. The majority of studies to date have been of plants and some invertebrate parthenogens, particularly aphids, and have not necessarily caught invasion at very early stages. Here we describe the early stages of an invasion by a Northern Hemisphere Hymenopteran model in three different land masses in the Southern Hemisphere. *Nematus oligospilus* Förster (Hymenoptera: Tenthredinidae), a sawfly feeding on willows (*Salix* spp.), was recently introduced to the Southern Hemisphere where it has become invasive and is strictly parthenogenetic. In this study, the number of *N. oligospilus* clones, their distribution in the landscape and on different willow hosts in South Africa, New Zealand and Australia were assessed using 25 microsatellite markers. Evidence is presented for the presence of two very common and widespread multilocus genotypes (MLGs) or ‘superclones’ dominating in the three countries. Rarer MLGs were closely related to the most widespread superclone; it is plausible that all *N. oligospilus* individuals were derived from a single clone. A few initial introductions to Australia and New Zealand seemed to have occurred. Our results point towards a separate introduction in Western Australia, potentially from South Africa. Rarer clones that were dominant locally putatively arose *in situ*, and might be locally favoured, or simply have not yet had time to spread. Data presented represent rare baseline data early in the invasion process for insights into the mechanisms that underlie the success of a global invader, and develop *Nematus oligospilus* as a valuable model to understand invasion genetics of clonal pests.

## Introduction

One of the major paradoxes of invasion biology is how species with very low genetic variation (and thus with expected low evolutionary potential) can still be successful invaders [1], [2], [3], [4]. As invaders usually arrive in very low numbers, genetic bottlenecks should reduce genetic diversity in the invasive range; for example: [5], [6], [7]. A number of hypotheses have been suggested to resolve this paradox. The first is that that genetic/genomic diversity is often not lost, but when it is, phenotypic variation may not be reduced due to plasticity [8]. In some cases, genetic diversity in the invasive range may increase owing to admixture between individuals from multiple sources [1], [9], [10]. Alternatively, it has been proposed that rapid evolution generates functional diversity and/or local adaptation [4], [11].

One commonly observed feature of invasive species is the tendency to become clonal in the invasive range, which has been suggested to enable invasive organisms to thrive in the face of low diversity [2]. Clonal organisms are more than four times over-represented among pest invertebrates [12]. Clonality has been highlighted as an important mechanism by which invaders with low genetic diversity succeed, and a meta-analysis uncovered the pattern that a disproportionate fraction of aquatic invaders with low diversity were asexual [2]. In addition to the demographic benefits of asexuality (notably not investing in males, and that a single individual can found a new colony), this mode of reproduction is generally impervious to inbreeding depression and fit genotypes are not broken up by sexual recombination [2]. Conversely, while lack of the ability to shuffle pools of alleles into new genotypic combinations is on face value disadvantageous, ecological traits of parthenogens with large population sizes nonetheless can diversify quickly by mutation, and rapid evolution is an increasingly recognized contributor to invasiveness [13], [14].

It is common for invasive organisms to transition to obligate or functional parthenogenesis; for example: [15], [16], [17], [18]. These transitions result in the unit of selection switching instantaneously from the gene to a co-inherited suite of genomes (nuclear, mitochondrial, symbionts) at least when the transitions are irreversible [19]. This has important implications for the diversity of genotypes and phenotypes, and the fitness consequence of their interactions with environmental conditions, and thus for control strategies [12]. An invasion that produces clones might be characterized by a large or small number of fixed clones, or some complex scenario of asexual/sexual interactions and recurrent generation of new clones. Natural selection is generally expected to act rapidly among clonal lineages, particularly when the number of individuals within lineages is large, the number of lineages small, and they persist over time [20]. Pre-adapted genotypes should be favoured, while strong selection against suboptimal genotypes should occur soon after introduction. Genotypes may be thought of as more or less ‘generalist’ or ‘specialist’ based on the breadth of their ecological niche and their environmental tolerances which can translate into their distributions in environmental space and time; both can lead to successful invasion [16], [21], [22], [23]. New phenotypes can arise following mutations and karyotypic changes [4], [11], [24] and it is of central interest in invasion genetics to what extent this is an effective part of invasion ability of clonal organisms.

A high proportion of studies on these topics have been of cyclic parthenogens; in particular, Hemiptera, and more specifically aphids, are extremely well-represented in pest species and studies of them [12], [25]. This important area of study requires strong models encompassing diverse organisms, with longitudinal studies starting early in invasions, ideally in multiple locations, applying highly-resolving genetic assays. A promising emerging model for understanding invasions of clonal organisms with low diversity is the willow sawfly *Nematus oligospilus* Förster (Hymenoptera: Tenthredinidae). This taxon has recently invaded the Southern Hemisphere [26]. It is native to Eurasia (from Ireland to the Himalayas) [27] and North America (from Alaska to Mexico) [28]. However, recent research points towards *N. oligospilus* representing a group of species and the native range of the invasive species is unknown [26], [29]. It was first detected in South America in 1980 [30], [31]. By 1993 it had spread to southern Africa [32], it reached New Zealand by 1997 [33] and was first detected in Australia (Canberra, Australian Capital Territory) in 2004 [34]. By 2009, it had established in most of southeast Australia (from South Australia to the New South Wales and Queensland border), Tasmania and southwestern Western Australia and was still undergoing expansion [35].

A recent study developed a high-resolution microsatellite assay and identified several multi-locus genotypes (MLGs) in *N. oligospilus* in its invasive range in the Southern Hemisphere [26]. In this paper, we seek to advance the understanding of the evolution of invasive parthenogens that diverge without sexual reproduction and genetic recombination. In addition, we identify *N. oligospilus* as an exciting model taxon of parthenogenetic invasion. Here we assess the distribution, genetic relatedness and host associations of *N. oligospilus* MLGs in South Africa, New Zealand and Australia from widespread, substantial sampling very early in the invasion process. Specifically, we address the following questions: 1/What clones of *N. oligospilus* are detectable in the invaded range? 2/What are their geographic and host-based distributions? 3/How closely related are they, and can their mode of origin be inferred? 4/Are there candidate ‘superclones’ and newly-evolved variants that would be valuable to monitor in time and space?

## Methods

### Field collection

In southeastern Australia, collections occurred in December 2007 and January 2008. The southern part of Western Australia was sampled in February 2010, while New Zealand was surveyed in February 2009. Individuals from South Africa were collected at one site in 2008 (n = 26). More than 1100 individuals were collected and genotyped from 21 sites in New Zealand and 69 sites in Australia (Table S1). All sites were on public land and sampling did not require permits. No endangered or protected species were sampled as part of this study.

Sites were selected on the basis of presence of willow taxa, and distance from other sites (usually 50 to 100 km apart). At each site, a single mature tree per willow taxon was sampled. Foliage was inspected visually for sawfly presence, and any sawfly stages were collected into 96% ethanol, to a maximum of 30 individuals per tree per site. Adults were excluded because of their high mobility, except for South Africa due to small sample size. Defoliation of willow trees (i.e. when foliage on any branch had been completely consumed irrespectively of the amount of defoliation) was noted. Willow species were identified using keys for southeastern Australia [36], [37], [38] and New Zealand (van Kraayenoord *et al.* 1995).

### DNA extraction and microsatellite analysis

The DNA of up to 10 *N. oligospilus* individuals was extracted per site per willow taxon using a salting-out DNA extraction protocol [39]. The type and amount of tissue used depended on the stage of the sawfly. For adults, DNA was extracted from the abdomen, while for larvae and pupae, the head and a portion of the thorax were used. Due to their small size, whole first-instar individuals were used.

Twenty-five microsatellite loci were used to identify multilocus genotypes (MLGs) that differ by one or more allele [26]. Fourteen of the loci were polymorphic in the Australian, New Zealand and South African samples, ten of which contributed to discrimination among genotypes. The loci that did not discriminate clones were nonetheless considered worth including (particularly given the convenience of simultaneous amplification of many loci in this system) for potential detection of rare/new variants since they are polymorphic in *N. oligospilus* in locations with males as well as females, and in other tenthredinid taxa [26]. Use of 25 loci should yield high resolution: previous studies have attributed clonal identity in introduced populations on the basis of many fewer loci, e.g. 5 to 7 microsatellite markers [40], [41]. Individuals bearing the same 25-locus genotype will be referred to as members of the same clone, while acknowledging that even mother-daughter pairs are likely to differ by at least a few point mutations somewhere in the genome. One primer of each primer pair was labelled with infra-red dyes IRD700 or IRD800 (LI-COR Biosciences). Using the Qiagen PCR multiplexing kit following the manufacturer's instructions, 12 and 13 loci were multiplexed in 10 µl reactions, with a maximum of seven loci per channel (Multiplex A: IRD700: WF2, WF11, WF15, WF21, WF26, WF27, WF40; IRD800: WF12, WF13, WF25, WF30, WF39, WF41. Multiplex B: IRD700: WF9, WF20, WF23, WF32, WF34, WF35, WF38, IRD800: WF6, WF10, WF14, WF16, WF37). PCR products were electrophoresed on 6% acrylamide gels in LI-COR Global IR2 two-dye DNA sequencers (models 4200 and 4300). Microsatellites were viewed in LI-COR software Saga Generation 2.0, and scored visually.

### Analyses

Genetic analyses were conducted according to sampling units based on country (Australia, New Zealand and South Africa), and within countries, regions separated by major land or water barriers between populations of *N. oligospilus*. The rationale for the choice of these geopolitical units is that their physical organization, economic activities and biosecurity implementation are likely to be dominant factors in the likelihood of introduction. Six regions were defined: two in New Zealand (the North and South Islands), three in Australia (southeastern Australia; southwestern Western Australia isolated from southeastern Australia by ∼1100 km of the Nullarbor Plain, and Tasmania isolated by ∼400 km from the nearest part of mainland Australia by the Bass Strait), and South Africa. Genotypic diversity (G/N) was calculated for each country and region, using the number of genotypes (G) divided by the number of individuals genotyped (N). Shannon-Weaver diversity index, allelic diversity, observed (Ho) and expected heterozygosity (He) were calculated in GenAlex 6.4 [42]. Shannon-Weaver diversity index [43] was expressed as *e^H^* to allow comparison based on the number of genotypes present in the populations. Departures from Hardy-Weinberg equilibrium (HWE), linkage disequilibrium and *F*-statistics were calculated and assessed with exact tests in Genepop 4.0 [44]. Pair-wise *F*_ST_ estimates between populations were calculated based on the 25 loci with combined statistical significance calculated according to Fisher's method as implemented in Genepop. HWE analyses were performed and expected and observed heterozygosity calculated initially for the full dataset, and then for a reduced dataset containing one individual per genotype to avoid distortions caused by multiple representatives of the same clonal genotype [40], [45].

**Unrooted neighbour-joining trees were constructed based on Cavalli-Sforza's chord measure of genetic distance** [46] **in Populations 1.2.32** (www.bioinformatics.org/~tryphon/populations/#ancre_fonctionnalites). One tree was built to depict the relationships among MLGs (inferred allele changes were mapped onto branches), and one tree depicted the population network on the basis of one individual per MLG per region. To assess genetic variance within and between countries and regions, a nested Analysis of molecular variance (AMOVA) was carried out with regions nested within countries in GenAlex 6.4 [42]. P-values were assessed with 999 random permutations.

## Results

### Detection and distribution of clones

**Figure 1:**
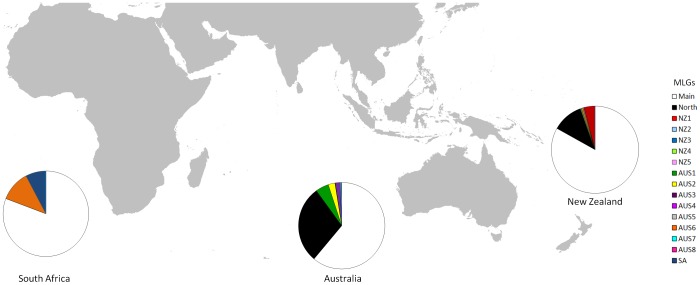
Frequency of multilocus genotypes (MLGs) of *Nematus oligospilus* in South Africa, Australia and New Zealand. Each circle represents a population and relative frequency of each MLG within the population. Each color represents a different MLG.

**Figure 2:**
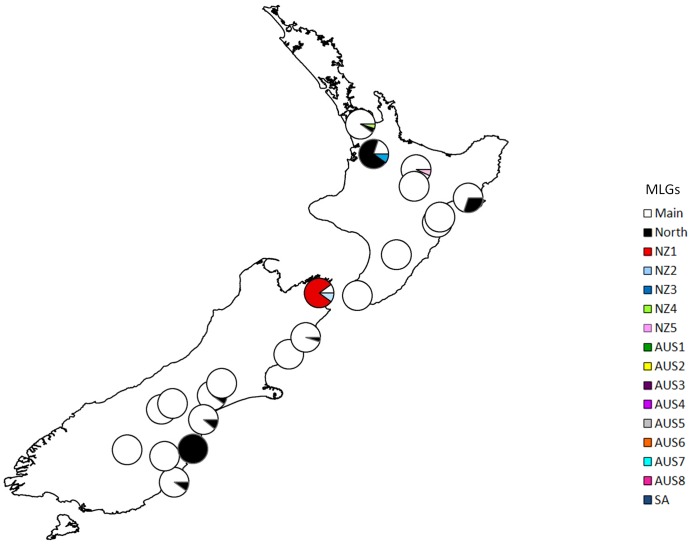
Frequency of multilocus genotypes (MLGs) of *Nematus oligospilus* in New Zealand. Each circle represents a location sampled and the relative frequency of each MLG within the site. Each color represents a different MLG.

**Figure 3:**
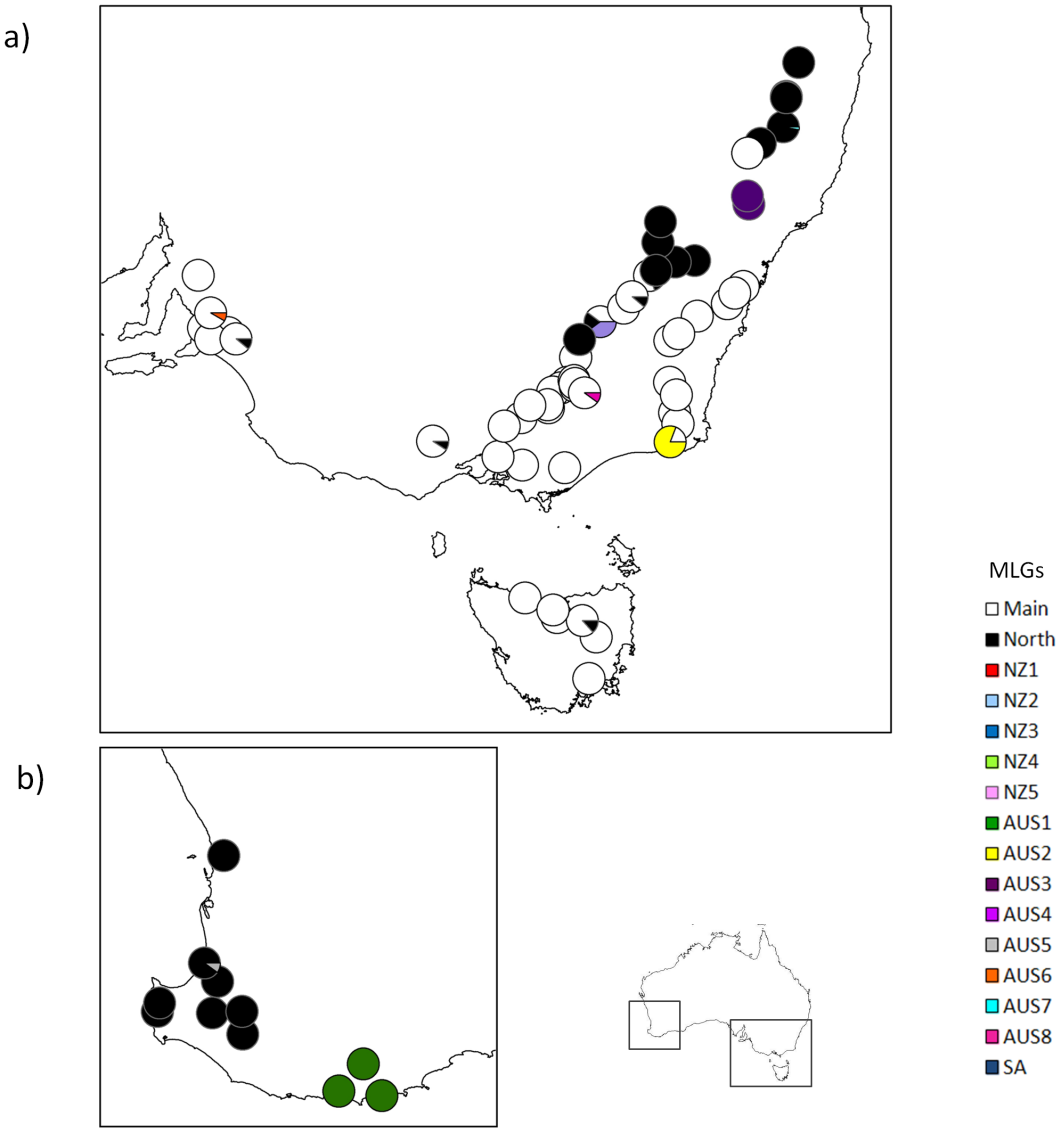
Frequency of multilocus genotypes (MLGs) of *Nematus oligospilus* in Australia. Each circle represents a location sampled and the relative frequency of each MLG within the site. Each color represents a different MLG. a) southeastern Australia and Tasmania b) Western Australia.

The combination of 25 microsatellite markers discerned 16 different MLGs in the Australasian and South African invaded range sampled (Table 1). Of the MLGs identified, ten were found in Australia, seven of which were unique to that continent. Similarly, seven MLGs were found in New Zealand, five of which were unique, while in South Africa, one of three clones was unique (Table 1, Fig. 1). The ‘Main clone’, the most widespread MLG, occurred over most sampled areas of Australia and New Zealand and was also present in South Africa (Figs. 1, 2, 3). It represented 65.7% of all individuals genotyped. It was, however, absent from Western Australia. The second most widespread MLG, the ‘North clone’ comprised 24.9% of individuals. It encompassed the northern part of southeastern Australia as well as most of Western Australia, although it was present in low proportion at several other locations (e.g. one site in Tasmania) (Fig. 3). It was also found on North and South Islands of New Zealand. It appeared to be spreading, because it was found in high proportions at some sites but smaller numbers in surrounding sites (Fig. 2). Although both clones were widespread, they rarely overlapped within a site.

**Table 1:** *Nematus oligospilus* multilocus genotypes (MLGs) with country, location and region (SE: Southeastern Australia, WA: Western Australia, TAS: Tasmania, SI: South Island, NI: North Island) where they occur, extent (widespread or localized), solely present (yes: single MLG present within sites, no: MLG found with other MLGs within sites), marker: the identity of the microsatellite locus showing differences when compared to Main MLG, and the frequency of each MLG.

MLG	Country	Region (location)	Extent	Solely present	Willow taxa	Marker	Frequency
Main	Australia,	SE, TAS	Widespread	Yes/No	All	N/A	0.657
	South Africa	-					
	New Zealand	NI, SI					
North	Australia	SE, WA, TAS	Widespread	Yes/No	All	WF2	0.249
	New Zealand	NI, SI					
Aus 1	Australia	WA (Albany)	Localized	Yes	*S.* X *sepulcralis*	WF2	0.039
					*S. babylonica*	WF40	
					*S. alba* var. *vitellina*		
Aus 2	Australia	SE (Cann River)	Localized	No	*S. fragilis/rubens*	WF2	0.020
					*S. matsudana* ‘tortuosa’		
					*S. alba* var. *vitellina*		
					*S. humboldtiana*		
					*S*. X *sepulcralis*		
					*S*. X *chrysocoma*		
Aus 3	Australia	SE (Scone)	Localized	Yes	*S*. X *sepulcralis*	WF30	0.015
Aus 4	Australia	SE (Junee)	Localized	No	*S. babylonica*	WF32	0.003
					*S. fragilis/rubens*		
Aus 5	Australia	WA (Bunbury)	Localized	No	*S. babylonica*	WF2	0.001
Aus 6	Australia	SE (Williamstown)	Localized	No	*S. fragilis/rubens*	WF35	0.003
	South Africa	-					
Aus 7	Australia	SE (Armidale)	Localized	No	*S. alba* var. *vitellina*	WF2	0.001
						WF9	
Aus 8	Australia	SE (Eskdale)	Localized	No	*S. fragilis/rubens*	WF20	0.001
SA	South Africa	-	n/a	No	n/a	WF40	0.003
NZ 1	New Zealand	SI (Picton)	Localized	No	*S. fragilis/rubens*	WF13	0.008
NZ 2	New Zealand	SI (Picton)	Localized	No	*S. fragilis/rubens*	WF13	0.001
NZ 3	New Zealand	NI (Hamilton)	Localized	No	*S. fragilis/rubens*	WF40	0.001
NZ 4	New Zealand	NI (Manurewa)	Localized	No	*S. alba* var. *vitellina*	WF25	0.001
NZ 5	New Zealand	NI (Rotorua)	Localized	No	*S. fragilis/rubens*	WF21	0.001

Other than ‘Main’ and ‘North’ clones, most MLGs were localized and had low frequencies. Three exceptions were clone Aus1, which was found in several sites in Western Australia around the port city of Albany and was unique to this region, and Aus2 and Aus3 which were common locally (Table 1, Fig. 2 and 3). Clone Aus2 occurred at one site, along with the Main clone. Clone Aus3 occurred at two isolated sites, where it was the only clone present (Table 1, Fig. 3). Other MLGs were usually represented by one individual per site. While two MLGs were found in more than one site (Aus2, and Aus 4), other MLGs were unique to a single site. Locally common MLGs were found on all willow taxa within a site. Few statements about distributions can be made for South Africa since all individuals were sampled at one site. However, one rare MLG was present in South Africa and South Australia (Aus6).

The two most widespread MLGs, ‘Main’ and ‘North’ clones were found on all willow taxa sampled showing no obvious host specialization. Similarly, locally abundant MLGs Aus1 and Aus2 were found on all the willow taxa present at the sites where they occurred. Only the ‘Main’ ‘North’ and Aus1 MLGs have been found at sites where willows suffered defoliation.

### Clone relatedness and genotypic diversity

**Figure 4:**
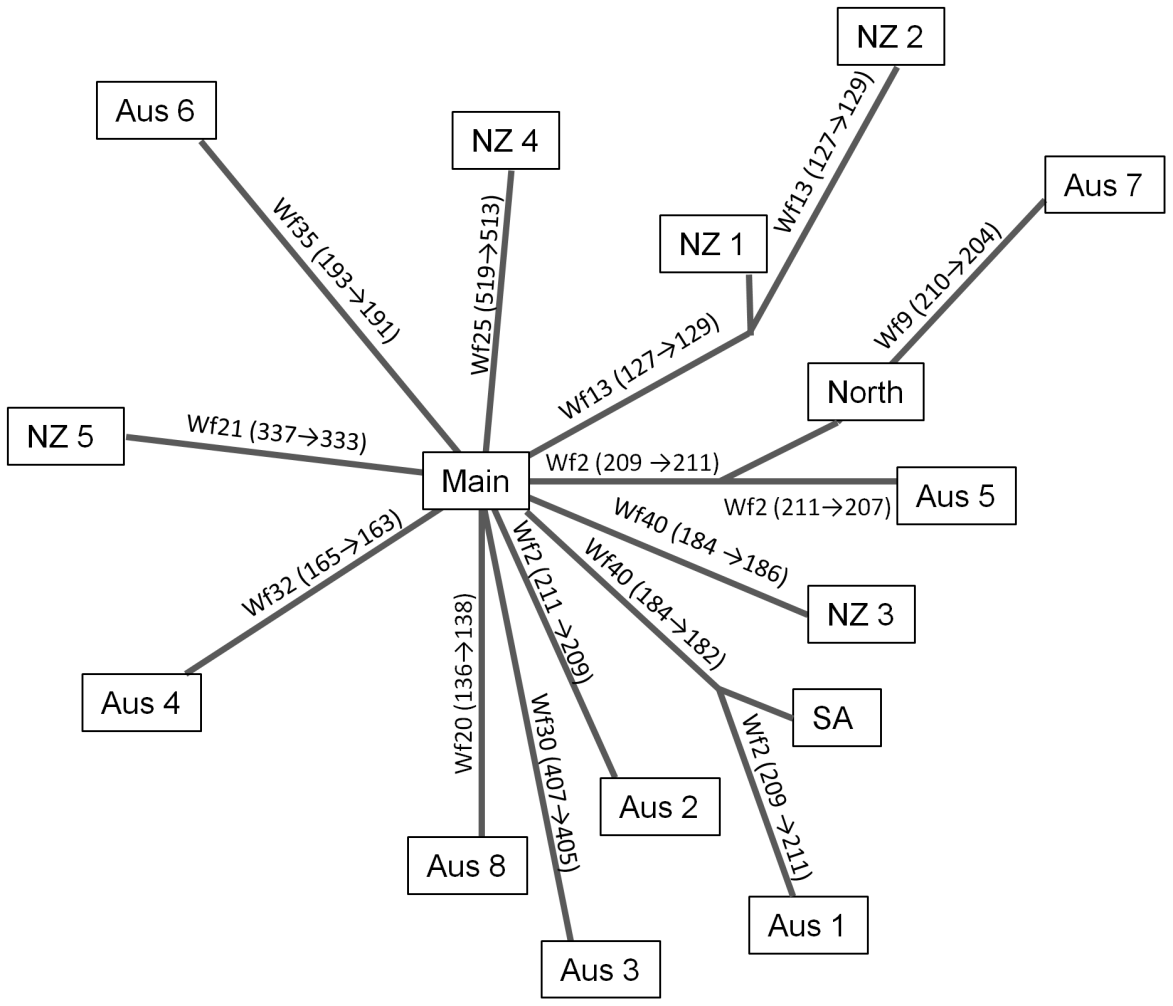
Unrooted neighbour-joining tree based on Cavalli-Sforza chord distance depicting relationships between different multilocus genotypes of *Nematus oligospilus*. Under the assumption that multilocus genotypes (MLGs) more interior in the network are the ancestors of more exterior ones on the same branch, inferred mutational changes are depicted on the branches, e.g. Wf21 (337→333) indicates that MLG NZ5 could have arisen most parsimoniously by a 4 base pair deletion of the Wf21^337^ allele from an ancestor with the genotype of Main MLG.

The 16 MLGs sampled were extremely similar at their 25-locus genotypes: they differed by a maximum of 3 alleles and 3 loci (Table 1). Although other interpretations are possible, the data are most parsimoniously consistent with most or all of the 15 MLGs being derived by mutation from the most common and widespread Main MLG. On an unrooted neighbour-joining tree of the MLGs, ‘Main’ is the most interior, and from it branch eight individual MLGs differing from Main each by a single allele difference (Fig. 4). Another three branches from Main each contain two or three MLGs linked by progressive single differences. One of the multi-MLG branches was limited in distribution to New Zealand (NZ1 and NZ2). The other two branches were not geographically restricted; one encompassed two localized MLGs found in Australia (Aus5 from Western Australia, Aus7 from Southeastern Australia) and also ‘North’, found in Australia and New Zealand, while the last multi-MLG branch spans Aus1 from Western Australia, and SA from South Africa. While the tree–building process did not allow reticulation, none is necessary to explain the data: MLG relationships can be depicted by simple sequential changes without homoplasy or ambiguity.

Overall, the three countries and regions had low genetic diversity based on genotypic diversity, *e^H^* and allelic diversity (Table 2). Shannon-Weaver diversity index was extremely similar for the three countries and regions, between 1.35 and 1.37. Observed heterozygosity and allelic diversity were equally low in the regions, respectively ranging from 0.42 to 0.44 and 1.48 to 1.68. There were frequent deviations from HWE in the direction of heterozygote excess, a common property of microsatellite data sets from obligate parthenogens proposed to diversify by mutation [47] (Table 2). The number of private alleles was low: New Zealand had three private alleles, while Australia had four and South Africa had none (the latter result likely contributed to by small sample size).

**Table 2:** Summary statistics for *Nematus oligospilus* by country (*N*: sample size, G: number of genotypes present, G/N: genotype diversity, *e^H^*: Shannon-Weaver diversity index, No. alleles: total no. of alleles over 25 loci, allelic diversity, Ho: observed heterozygosity, He: expected heterozygosity, *P* excess: heterozygosity excess, n/s:non-significant (p>0.05), * : significant (p<0.05) (He, Ho, *P* and *F*_IS_ were calculated using one individual per genotype).

	Australia				New Zealand			South Africa
	WA	SE	Tas	All	North	South	All	
*N*	99	760	53	911	100	119	219	26
No. genotypes	3	8	2	10	5	4	7	3
G/N	0.03	0.01	0.04	0.01	0.05	0.03	0.03	0.11
*e^H^*	1.36	1.36	1.35	1.36	1.36	1.37	1.36	1.37
No. alleles	37	40	37	43	38	37	39	38
Allelic diversity	1.48	1.60	1.48	1.68	1.52	1.48	1.56	1.52
Private alleles	1	3	0	4	2	1	3	0
Ho	0.440	0.420	0.420	0.420	0.440	0.440	0.440	0.440
He	0.233	0.221	0.215	0.229	0.242	0.224	0.240	0.229
*P* excess	n/s	*	n/s	*	*	n/s	*	n/s
Multilocus *F*_IS_	−0.879	−0.820	−0.909	−0.814	−0.886	−0.808	−0.805	−0.886

### Low genetic differentiation among countries and regions

**Figure 5:**
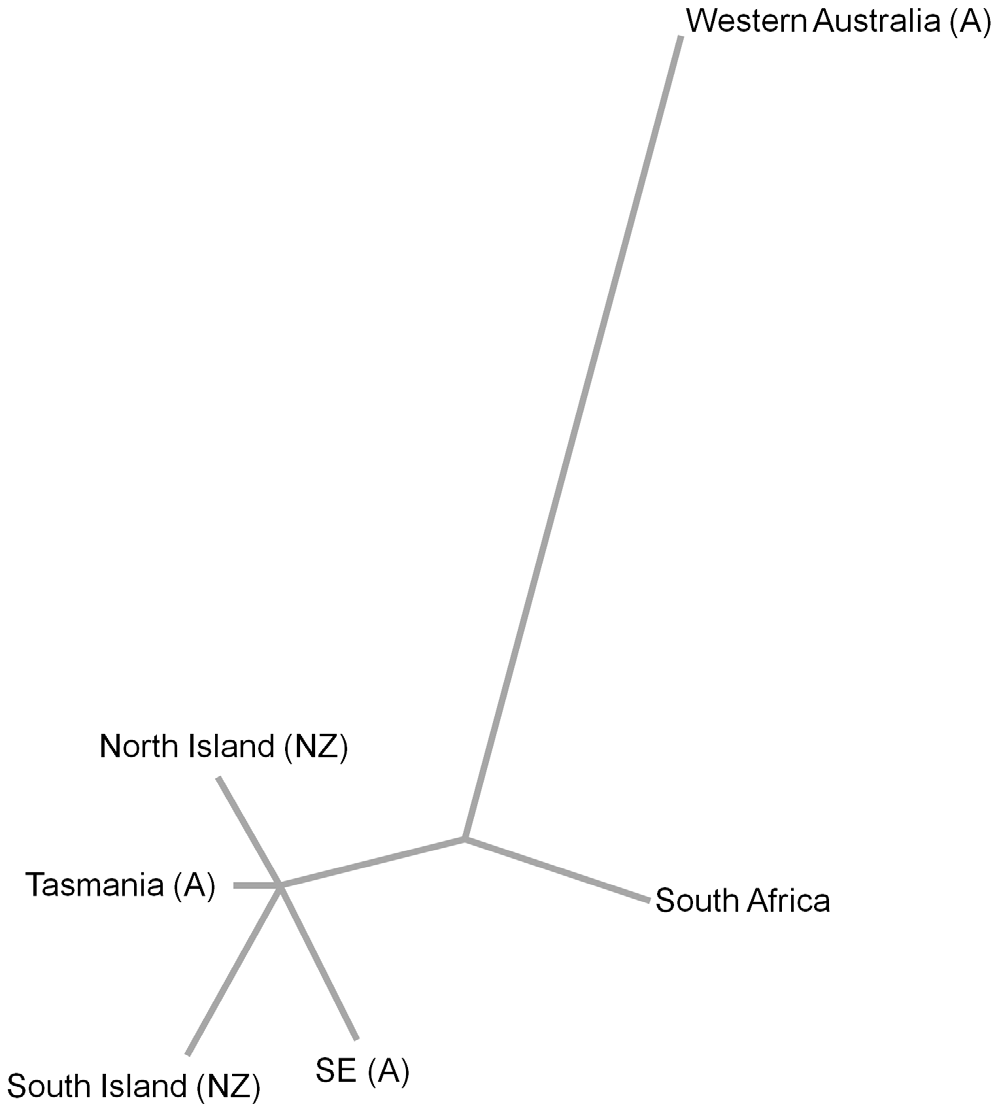
Unrooted neighbour-joining tree based on Cavalli-Sforza chord distance depicting relationships between different regions where *Nematus oligospilus* occurs in the Southern Hemisphere (SE: Southeastern, A: Australia, NZ: New Zealand) on the basis of the multilocus genotypes (MLGs) present in each region.

*F*_ST_s for all country or region pairs were low, ranging from less than 0.001 to 0.05. All pairwise comparisons showed that sampling locations (countries or regions) did not differ significantly in allele frequencies (p>0.05). According to the AMOVA, most of the molecular variance, 88%, was attributed to within-region effect, while only 12% was attributed to between-region effects and less than 1% to between-country effects (Table 3). The population tree placed the regions into two groups. The main grouping included southeastern Australia, Tasmania, the North and South Islands of New Zealand, with South Africa very slightly outside that group, and with Western Australia placed on a long branch slightly more allied to South Africa (Fig. 5), presumably owing to the close relationship of Western Australian-limited MLG Aus 1 and South Africa-limited SA.

**Table 3:** Results from the nested Analysis of Molecular Variance (regions nested within countries) (df: degree of freedom, SS: sum of squares, MS mean squares, Est. Variance: Estimate of variance, % variation: percentage of total variation.

Source	df	SS	MS	Est. variance	% variation	*P*
**Among countries**	2	66.006	33.003	<0.001	0%	1.0
**Among regions**	3	128.705	42.902	0.325	12%	0.001
**Within regions**	1151	2770.058	2.407	2.407	88%	0.001

***P*** **: P-value (calculated from 999 permutations).**

## Discussion

### Clone distribution and relatedness

*Nematus oligospilus* is a very successful parthenogenetic invasive organism with low genotypic variation, and two extremely successful genotypes. The ‘Main’ MLG has colonized three continents and was present in all regions studied except Western Australia, while the ‘North’ MLG was widespread in Australia, and New Zealand. They were both found on all willow taxa present, indicating little host specialization. This, coupled with their wide geographic distribution and the very high proportion of individuals sampled belonging to them, means that they fit published definitions of ‘superclones’: genotypes dominating populations over large areas; for example: [41], [48]. Other clonal invasive organisms also have only a few widespread genotypes in their introduced range. Examples are relatively common in plants, with many cases of one or a few dominant clones in their invasive populations; for example: [22], [49] and in some invertebrates such as aphids [21], [40], [41], [48]. Our data are unusual in that they are particularly detailed (25 loci compared to as few as five microsatellites in similar studies [41]) and samples animals from early in the invasion process. The widespread distribution of the two very common MLGs, their wide host range and the fact they were found singly at sites where willows had been defoliated may indicate that characteristics of these clones contribute to early invasion success. Our study expands the published literature to encompass highly-resolving genotypic patterns in a representative of the family Tenthredinidae, a dominant component of the Hymenopteran parthenogenetic fauna [12].

Clones were closely related, suggesting that the number of introductions into the Southern Hemisphere has been few, except perhaps if they came from a stock of unusually limited diversity. We infer on the basis of the network of MLGs, with support from geographic and numerical distributions, that many of the clones are descended by mutation from an early invader (very likely the ‘Main’ clone). For example, some of the rarer clones seem to have arisen *in situ*, as they occurred in very low proportions at sites where a common MLG predominated and differed from it at only a single allele at a single locus. Following introduction in new environments, clonal lineage changes can occur through mutations [4] as observed in other studies of invertebrates and plants [11], [49], [50]. The dominance of localized MLGs could be due to being favoured by environmental conditions. Conclusive determination of whether these MLGs were introduced separately or have arisen *in situ* would require further work, but given their geographic abundance distributions and heterozygous excess (a characteristic of mutational divergence in clones, [47]), we currently favour the latter explanation. Possibly, rarer MLGs have been or will be favoured by environmental conditions; such a process could explain the presence of some localized MLGs dominating some site. However, the present paper presents one of the few cases globally where such a process could be inferred, and so *N. oligospilus* and the newly-available genetic tools for it offer many possibilities to test specific hypotheses concerning the success of clonal invaders [51], [52].

### Host association

Host plants can provide selection pressures that lead to specialization of herbivore populations [53], [54]. While clonal specialization has been observed in some invading aphids [16], [45], the widespread MLGs of *N. oligospilus* did not depend on association with specific willow taxa, indicating that this species may be a host generalist. However, in the field, when several willow hosts are present, population densities of *N. oligospilus* are constantly higher on some host taxa than others [35]. The less favoured hosts may be more challenging and exert higher selection on *N. oligospilus* and increase the chance for mutations to spread. In a study on *Sitobion* aphids, although no host specialization was found, rarer clones had enhanced performance on the most chemically defended host plants, and it was hypothesized that rarer clones were relatively favoured partly because of the presence of chemical defences [55]. Due to the low numbers of the rarer clones, this could not be assessed in this study, but warrants future investigation.

### Introduction and dispersal in the landscape

The native range source of the *N. oligospilus* that have invaded the Southern Hemisphere has not yet been resolved, and genetic data can contribute to clarification of relationships of these insects around the world [29], [35]. The modest number of extremely closely-related clones, all derivable from the ‘Main’ clone by very few mutations, indicates a small number of introductions from a single source. All Australasian and the limited number of South African individuals assayed are possibly derived from a single founding clone. However within the sampled invaded range, multiple introductions may have occurred. For example, the unique clone in Western Australia (‘Aus1’) is more closely allied to a South African MLG than it is to Australian clones (although the relationships are all extremely close). Thus Aus1 may represent an independent introduction into Australia, quite possibly from South Africa, although a more thorough sampling of the African continent is required. Many unwanted introductions to Western Australia have occurred from South Africa, including many plants and invertebrates such as the African black beetle, now considered an important pest [56], [57]. An independent introduction into Western Australia is supported by reports of sawflies first being seen there five years later than in the rest of Australia (2004) (F. Ede, unpublished).

Sawflies in general are not strong flyers [58]. Therefore, self-dispersal could occur over limited areas but is unlikely over large water- and land-barriers. For example, it is highly unlikely that *N. oligospilus* flew from South Africa to Australasia, although we cannot rule out transport by wind. Similarly, it seems improbable that ‘North’ MLG found in southeastern Australia and Western Australia dispersed unassisted across 1100 km of the Nullarbor Plain (arid habitat lacking willow hosts), or that the single individual of the ‘North’ clone sampled in Tasmania self-dispersed to the location of its capture close to Launceston airport. Most of the dispersal seen between regions is more likely due to human-mediated jump dispersal, which is also a major cause of long-dispersal in many other invasive species; for example: [59], [60]. When ready to pupate, larvae move or drop to the ground, spinning cocoons on vegetation or in soil litter. In high infestation areas, cocoons can be found on any surface available including outdoor furniture and cars (Caron, pers. obs.). Cocoons could therefore easily be transported inadvertently for long distances. Furthermore, in at least a few cases, people have moved sawflies deliberately in the hope of controlling willow infestations (F. Ede, pers. obs.). Human-related dispersal of invasive organisms is common and observed in other introduced sawflies [61] and other parthenogenetic pests such as grape phylloxera (*Daktulosphaira vitifoliae* Fitch), in which dispersal among localities is due solely to human-mediated movements of infected plants [62]. However, self-dispersal plausibly explains much of the spread of the Main and North MLG at smaller spatial scales within southeastern Australia and New Zealand.

### Conclusions

Two dominant MLGs (or superclones) of *N. oligospilus* occur in the Southern Hemisphere invasive range. Empirically these superclones are highly successful invaders, comprising most of the spread of *N. oligospilus* through the colonized range. Whether this success persists over longer periods remains to be tested. Clones with broader preferences and tolerances may be better at colonizing new areas, but may not be as good at competing [63]. Thus we expect invasions to be dynamic in their demogenetics: for example, one or a few genetic variants can be closely associated with dispersal ability but antagonistic to reproductive success [64]. For *N. oligospilus*, new MLGs seem to have arisen *in situ* within the Southern Hemisphere. Although two main MLGs now dominate, new and rare MLGs may be favoured subsequently, following changes in environmental conditions and as non-equilibria such as founder effects dissipate [3]. Furthermore, additional introductions of distinctive MLGs to the introduced population could provide new impetus to invasion, as seen with the multiple introductions of specialised clones of the pea aphid [16]. While few introductions seem to have occurred in the present case, the extreme success of *N. oligospilus* in the Southern Hemisphere highlights the importance of biosecurity. The introduction of a single individual capable of reproducing asexually and pre-adapted to the conditions of the new environment could lead to a successful invasion.

We propose that *Nematus oligospilus* is a valuable model in invasion genetics. Data and tools have been established early during invasion of significant sections of the globe. We encourage repeated and broad sampling of the invaded range and potential native range to help infer the mode and location of generation of MLGs. Coupled with detailed studies of the attributes of tractable individual clones, this can greatly add to the understanding of invasion genetics of clonal pests.

## Supporting Information

Table S1 Sites and willow taxa surveyed. N: number of *Nematus oligospilus* genotyped.
(DOCX)
