# Supplementary material for: Unravelling the Paradox of Loss of Genetic Variation during Invasion: Superclones May Explain the Success of a Clonal Invader
Source: PLoS One. 2014 Jun 10;9(6):e97744. doi: 10.1371/journal.pone.0097744 (PMC4051638; doi:10.1371/journal.pone.0097744)
Supplement: Table S1 — Sites and willow taxa surveyed. N: number of Nematus oligospilus genotyped. (DOCX) [file pone.0097744.s001.docx]

Table S1. Sites and willow taxa surveyed. N: number of *Nematus oligospilus* genotyped.

| **Country** | **State** | **Location** | **Latitude** | **Longitude** | **Willow taxa** | **N** |
| --- | --- | --- | --- | --- | --- | --- |
| Australia | Australian | Canberra | -35.29833 | 149.14306 | S. alba var. vitellina | 5 |
|  | Capital |  |  |  | S. fragilis/rubens | 10 |
|  | Territory |  |  |  | S. sepulcralis var. sepulcralis | 17 |
|  | New South | Armidale | -30.51139 | 151.66944 | S. alba var. vitellina | 10 |
|  | Wales |  |  |  | S. fragilis/rubens | 10 |
|  |  |  |  |  | S. matsudana x alba | 10 |
|  |  |  |  |  | S. sepulcralis var. sepulcralis | 9 |
|  |  | Bathurst | -33.50417 | 149.69333 | S. fragilis/rubens | 10 |
|  |  |  | -33.43306 | 149.33167 | S. reichardtii | 10 |
|  |  | Bendemeer | -30.88083 | 151.15806 | S. alba var. vitellina | 9 |
|  |  |  |  |  | S. babylonica | 7 |
|  |  |  |  |  | S. fragilis/rubens | 9 |
|  |  |  |  |  | S. matsudana 'tortuosa' | 3 |
|  |  | Blayney | -33.53944 | 149.25583 | S. alba var. vitellina | 7 |
|  |  |  | -33.53694 | 149.25667 | S. fragilis/rubens | 9 |
|  |  |  |  |  | S. sepulcralis var. chrysocoma | 10 |
|  |  | Bombala | -36.90917 | 149.24306 | S. fragilis/rubens | 10 |
|  |  | Bondi | -37.16028 | 149.31944 | S. fragilis/rubens | 9 |
|  |  | Campbell Town | -34.08389 | 150.77556 | S. sepulcralis var. sepulcralis | 9 |
|  |  | Cooma | -36.23111 | 149.13333 | S. matsudana 'tortuosa' | 9 |
|  |  | Cowra | -33.83750 | 148.68611 | S. fragilis/rubens | 5 |
|  |  | Culcairn | -35.67111 | 147.03556 | S. sepulcralis | 9 |
|  |  | Glen Innes | -29.82917 | 151.73833 | S. fragilis/rubens | 10 |
|  |  | Glencoe | -29.85667 | 151.73778 | S. fragilis/rubens | 6 |
|  |  | Goulburn | -34.74722 | 149.74972 | S. babylonica | 10 |
|  |  | Junee | -34.87222 | 147.58222 | S. babylonica | 7 |
|  |  | Mittagong | -34.46778 | 150.42111 | S. alba var. vitellina | 10 |
|  |  | Molong | -33.09861 | 148.87222 | S. fragilis/rubens | 9 |
|  |  | Musswellbrook | -32.24056 | 150.90278 | S. humboldtiana | 9 |
|  |  | Nimmitabel | -36.51389 | 149.28306 | S. alba X | 10 |
|  |  |  |  |  | S. sepulcralis var. chrysocoma | 10 |
|  |  | Scone | -32.05861 | 150.87056 | S. sepulcralis var. sepulcralis | 8 |
|  |  | Sutton | -35.14361 | 149.33611 | S. alba var. vitellina | 9 |
|  |  |  |  |  | S. babylonica X | 10 |
|  |  |  |  |  | S. fragilis/rubens | 7 |
|  |  | Tahmoor | -34.23222 | 150.58694 | S. babylonica | 10 |
|  |  | Tamworth | -31.09583 | 150.87889 | S. sepulcralis var. sepulcralis | 5 |
|  |  | Tenterfield | -29.06833 | 152.01361 | S. fragilis/rubens | 9 |
|  |  | The Rock | -35.27111 | 147.12250 | S. babylonica | 10 |
|  |  |  |  |  | S. fragilis/rubens | 10 |
|  |  | Wallendbeen | -34.56667 | 148.10639 | S. babylonica | 5 |
|  |  | Wellington | -32.54306 | 148.93222 | S. babylonica | 10 |
|  |  |  |  |  | S. fragilis/rubens 1 | 5 |
|  |  |  |  |  | S. fragilis/rubens 2 | 10 |
|  |  | Woodstock | -33.72167 | 148.82889 | S. babylonica | 10 |
|  |  | Young | -34.31472 | 148.29417 | S. matsudana 'tortuosa' | 5 |
|  | South | Clare | -33.78528 | 138.59194 | S. fragilis/rubens | 3 |
|  | Australia |  | -33.83333 | 138.61056 | S. sepulcralis var. sepulcralis | 10 |
|  |  | Little Hampton | -35.05278 | 138.85611 | S. sepulcralis var. chrysocoma | 1 |
|  |  | Lobethal | -34.89806 | 138.87472 | S. babylonica | 1 |
|  |  |  |  |  | S. fragilis/rubens | 9 |
|  |  | Mount Barker | -35.06722 | 138.85556 | S. alba var. vitellina | 3 |
|  |  |  |  |  | S. matsudana 'tortuosa' | 2 |
|  |  | Murray Bridge | -35.11556 | 139.27778 | S. babylonica | 4 |
|  |  |  |  |  | S. fragilis/rubens | 3 |
|  |  |  |  |  | S. sepulcralis var. sepulcralis | 7 |
|  |  | Stirling | -35.01389 | 138.73194 | S. fragilis/rubens | 5 |
|  |  |  |  |  | S. sepulcralis var. sepulcralis | 5 |
|  |  | Strathalbyn | -35.25861 | 138.88694 | S. sepulcralis var. sepulcralis | 10 |
|  |  | Tailem Bend | -35.25778 | 139.45444 | S. sepulcralis var. sepulcralis | 10 |
|  |  | Williamstown | -34.67778 | 138.88583 | S. babylonica | 3 |
|  |  |  |  |  | S. fragilis/rubens | 10 |
|  | Victoria | Ballarat | -37.54861 | 143.84556 | S. alba var. vitellina | 4 |
|  |  |  |  |  | S. fragilis/rubens | 5 |
|  |  |  |  |  | S. matsudana 'tortuosa' | 8 |
|  |  | Baranduda | -36.23194 | 146.84556 | S. babylonica | 7 |
|  |  |  |  |  | S. fragilis/rubens | 7 |
|  |  | Beechworth | -36.35722 | 146.69583 | S. cinerea | 5 |
|  |  | Bonnie Doon | -37.02472 | 145.81194 | S. babylonica | 10 |
|  |  | Cann River | -37.56667 | 149.14639 | S. alba var. vitellina | 2 |
|  |  |  |  |  | S. fragilis/rubens | 10 |
|  |  |  |  |  | S. humboldtiana | 3 |
|  |  |  |  |  | S. sepulcralis var. chrysocoma | 2 |
|  |  |  |  |  | S. sepulcralis var. sepulcralis | 9 |
|  |  | Cheshunt | -36.79806 | 146.42611 | S. fragilis/rubens | 8 |
|  |  | Everton | -36.44500 | 146.52278 | S. alba var. vitellina | 10 |
|  |  |  |  |  | S. babylonica | 10 |
|  |  |  |  |  | S. fragilis/rubens | 10 |
|  |  |  |  |  | S. nigra | 10 |
|  |  |  |  |  | S. purpurea | 10 |
|  |  | Finns Creek | -36.19194 | 147.00028 | S. nigra | 8 |
|  |  | Kergunyah | -36.32333 | 147.02972 | S. babylonica | 10 |
|  |  |  |  |  | S. fragilis/rubens | 9 |
|  |  | King Valley | -36.73167 | 146.40639 | S. alba var. vitellina | 10 |
|  |  | Robin Hood | -38.08528 | 145.84500 | S. babylonica | 10 |
|  |  |  |  |  | S. fragilis/rubens | 15 |
|  |  | Rosedale | -38.14194 | 146.79139 | S. babylonica | 10 |
|  |  |  |  |  | S. sepulcralis var. chrysocoma | 2 |
|  |  | Swanpool | -36.74556 | 146.01139 | S. fragilis/rubens | 10 |
|  |  | Tangambalanga | -36.25444 | 147.01833 | S. babylonica | 10 |
|  |  |  |  |  | S. fragilis/rubens | 10 |
|  |  |  |  |  | S. humboldtiana | 10 |
|  |  | Ferntree Gully | -37.89694 | 145.30194 | S. sepulcralis var. sepulcralis | 8 |
|  |  | Yea | -37.21250 | 145.44083 | S. fragilis/rubens | 5 |
|  | Tasmania | Burnie | -41.05694 | 145.90556 | S. alba var. vitellina | 7 |
|  |  | Campbell Town | -41.93306 | 147.49333 | S. fragilis/rubens | 10 |
|  |  |  |  |  | S. sepulcralis var. chrysocoma | 9 |
|  |  | Elizabeth Town | -41.49889 | 146.61917 | S. alba var. vitellina | 2 |
|  |  | Hobart | -42.86472 | 147.33306 | S. matsudana 'tortuosa' | 11 |
|  |  | Perth | -41.56444 | 147.18278 | S. fragilis/rubens | 5 |
|  |  |  |  |  | S. sepulcralis var. sepulcralis | 9 |
|  | Western | Albany | -35.01546 | 117.89429 | S. alba var. Vitellina | 5 |
|  | Australia |  |  |  | S. babylonica | 10 |
|  |  |  |  |  | S. babylonica X | 10 |
|  |  |  |  |  | S. sepulcralis var. sepulcralis | 10 |
|  |  | Bridgetown | -33.95626 | 116.13747 | S. babylonica | 8 |
|  |  | Bunbury | -33.34576 | 115.66639 | S. babylonica | 10 |
|  |  | Cowaramup | -33.85145 | 115.10181 | S. matsudana 'tortuosa' | 7 |
|  |  | Denmark | -34.95844 | 117.35320 | S. babylonica | 10 |
|  |  | Manjimup | -34.24313 | 116.14709 | S. babylonica | 1 |
|  |  |  |  |  | S. humboldtiana | 9 |
|  |  | Margaret River | -33.95662 | 115.07459 | S. babylonica | 5 |
|  |  | Perth | -31.94415 | 115.80994 | S. babylonica 2 | 4 |
|  |  |  |  |  | S. babylonica3 | 10 |
| New Zealand | North | Hamilton | -37.98250 | 175.31639 | S. fragilis/rubens | 10 |
|  | Island | Hastings | -39.63183 | 176.84719 | S. sepulcralis var. sepulcralis | 10 |
|  |  | Manurewa | -37.25917 | 174.99889 | S. alba var. vitellina | 10 |
|  |  |  |  |  | S. sepulcralis var. chrysocoma | 10 |
|  |  | Napier | -39.50397 | 176.90875 | S. matsudana 'tortuosa' | 5 |
|  |  | Palmerston North | -40.41083 | 175.86250 | S. fragilis/rubens | 10 |
|  |  | Picton | -41.33472 | 174.00333 | S. fragilis/rubens | 10 |
|  |  | Rotorua | -38.35611 | 176.33750 | S. fragilis/rubens | 10 |
|  |  |  |  |  | S. matsudana 'tortuosa' | 5 |
|  |  | Taupo | -38.76000 | 176.29056 | S. fragilis/rubens | 10 |
|  |  | Wairoa | -39.04028 | 177.59000 | S. fragilis/rubens | 10 |
|  |  | Wellington | -41.39806 | 174.92583 | S. matsudana 'tortuosa' | 10 |
|  | South | Cheviot | -42.81509 | 173.27492 | S. fragilis/rubens | 10 |
|  | Island | Cromwell | -45.12250 | 169.38278 | S. matsudana 'tortuosa' X | 10 |
|  |  | Dunedin | -45.90583 | 170.51500 | S. sepulcralis var. chrysocoma | 10 |
|  |  | Kaikoura | -42.40745 | 173.68022 | S. fragilis/rubens | 10 |
|  |  |  |  |  | S. matsudana 'tortuosa' | 10 |
|  |  | Mayfield | -43.80917 | 171.42972 | S. fragilis/rubens | 10 |
|  |  | Oamaru | -45.10056 | 170.96816 | S. matsudana 'tortuosa' | 10 |
|  |  | Pukaki | -44.14667 | 170.21056 | S. sepulcralis var. chrysocoma | 5 |
|  |  | Rakaia Gorge | -44.14667 | 170.21056 | S. alba var. vitellina | 1 |
|  |  |  |  |  | S. fragilis/rubens | 4 |
|  |  |  |  |  | S. reichardtii | 5 |
|  |  | Ranfurly | -45.27639 | 170.28667 | S. sepulcralis var. chrysocoma | 9 |
|  |  | Tekapo | -44.00444 | 170.47750 | S. fragilis/rubens | 5 |
|  |  | Timaru | -44.39306 | 171.22028 | S. sepulcralis var. chrysocoma | 10 |
| South Africa | Free State | Bethlehem |  |  |  | 26 |
